# Covid-19 Presenting as Acute Limb Ischemia

**DOI:** 10.7759/cureus.9344

**Published:** 2020-07-22

**Authors:** Balraj Singh, Parminder Kaur, Nora Ajdir, Sachin Gupta, Michael Maroules

**Affiliations:** 1 Hematology/Oncology, Saint Joseph's University Medical Center, Paterson, USA; 2 Cardiology, Saint Joseph's University Medical Center, Paterson, USA; 3 Internal Medicine, Saint Joseph's University Medical Center, Paterson, USA; 4 Internal Medicine, Reading Hospital, West Reading, USA

**Keywords:** covid-19, acute limb ischemia, arterial thrombosis, thrombosis, sars-cov-2 (severe acute respiratory syndrome coronavirus 2

## Abstract

Coronavirus disease-2019 (COVID-19) is caused by severe acute respiratory syndrome coronavirus 2 (SARS-CoV-2) and represents a potentially fatal disease. COVID-19 is associated with a hypercoagulable state leading to increased incidence of venous thromboembolism. Arterial thrombosis has been reported, but the prevalence is not known. Herein, we report an unusual presentation of a 77-year-old male who presented with dyspnea and pain in left leg and was found to have acute limb ischemia. Our case adds to the limited literature regarding arterial thrombosis in COVID-19.

## Introduction

At the end of 2019, a novel coronavirus was identified as the cause of a cluster of pneumonia cases in Wuhan, a city in the Hubei Province of China. On March 11, 2020, WHO declared coronavirus disease 2019 (COVID-19) as pandemic [1]. Droplets and contact are the main means of transmission [2]. Patients with severe acute respiratory syndrome coronavirus 2 (SARS-CoV-2) infection mainly present with upper and lower respiratory tract symptoms, with complications related to cytokine storm syndrome and acute respiratory distress syndrome (ARDS) [3]. We report an unusual presentation of a 77-year-old male who presented with dyspnea and pain, swelling in the left leg, and was found to have acute limb ischemia (ALI). 

## Case presentation

A 77-year-old male patient presented to emergency department with complaints of shortness of breath and pain, discoloration, and swelling of the left leg. Vital signs on presentation were heart rate 110 per minute, blood pressure 155/85 mmHg, oxygen saturation 96% on room air, and temperature 36 degree Celsius. The patient had bilateral crackles on lung exam and absent left dorsalis pedis and posterior tibial pulses and foot was swollen, discolored, and cold. Electrocardiogram showed sinus tachycardia 110 per minute.

**Figure 1:**
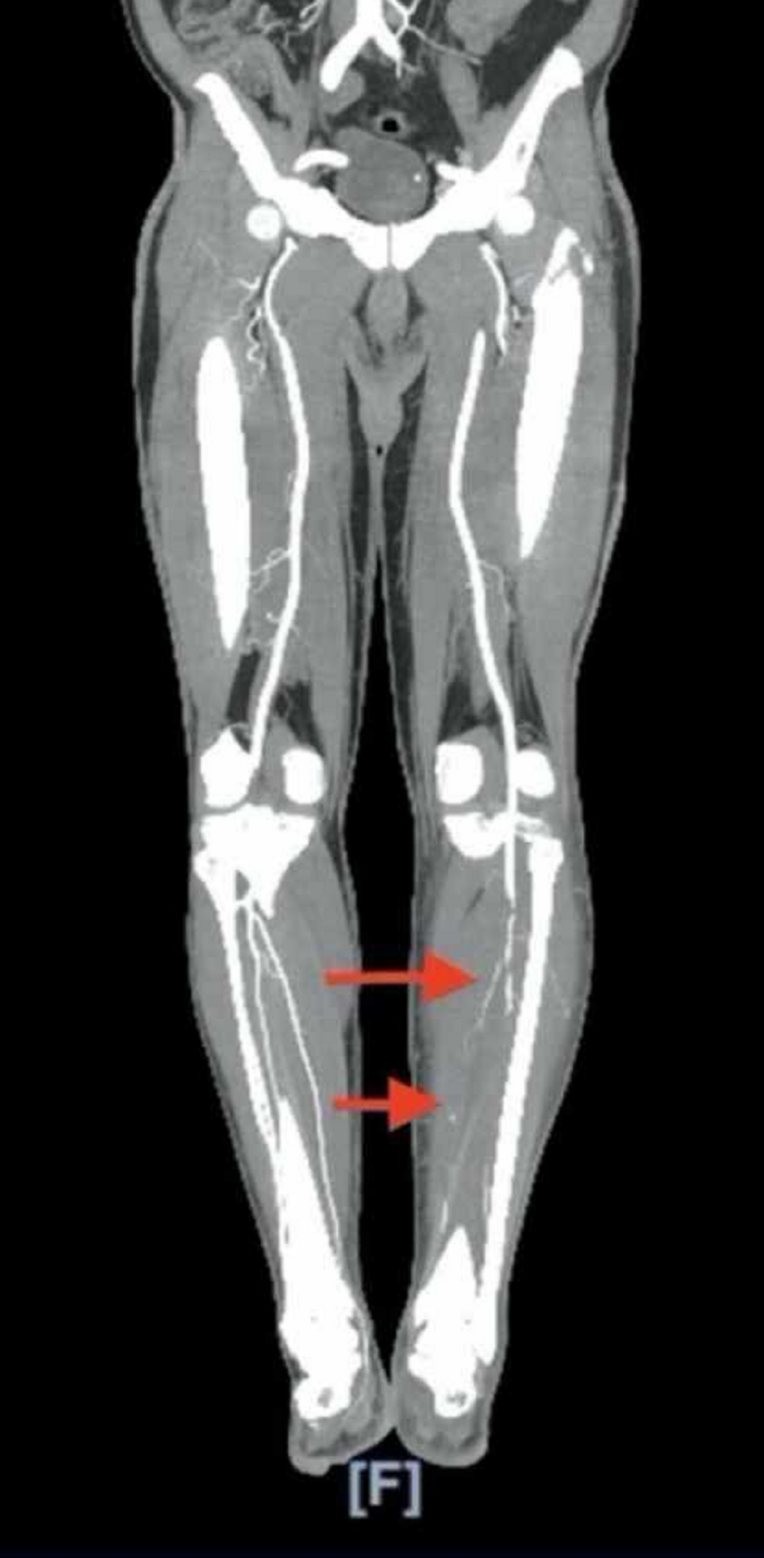
CT angiography of aorta with iliofemoral runoff showing occluded left anterior tibial artery and left peroneal artery.

On initial laboratory evaluation, the following values were noted: hemoglobin 12.1 g/dl (reference: 12-16 g/dl), hematocrit 38.2% (reference: 36%-46%), white blood cells 41 K/mm^3^ (reference: 4.5-11 K/mm^3^), platelets 534 K/mm^3^ (reference: 140-440 K/mm^3^), potassium 4.1 meq/L (reference: 3.5-5 meq/L), glucose 126 mg/dl (reference: 70-105 mg/dl), creatinine 0.61 mg/dl (reference: 0.6-1.30 mg/dl), blood urea nitrogen 8 mg/dl (reference: 7-23 mg/dl), lactic acid 1.1 mmol/L (reference: 0.5-2.2 mmol/L), troponin 0.136 ng/ml (reference: less than 0.03 ng/ml), D-dimer 2.77 (reference: less than 0.5), prothrombin time 14.8 seconds (reference: 12.2-14.9 seconds), international normalized ratio (INR) 1.2 (reference: less than 1), partial thromboplastin time 39.8 seconds (reference: 21.3-35.1 seconds), lactate dehydrogenase (LDH) 392 U/L (reference: 140-271 U/L), C-reactive protein (CRP) 301 mg/L (reference: less than 10 mg/L), ferritin 1,396 ng/mL (reference: 12-300 ng/ml), procalcitonin 0.60 ng/ml (reference: less than 2 ng/ml), aspartate transaminase 134 U/L (reference: 13-39 U/L), calcium 8.5 mg/dl (reference: 8.6-10.3 mg/dl), and albumin 2.7 mg/dl (reference: 3.5-5.0 mg/dl). Chest X ray showed bilateral hazy infiltrates. CT angiography of the abdomen and aorta with Iliofemoral runoff showed thrombus within the mid left superficial femoral artery and occluded left anterior tibial artery, left posterior tibial artery, and left peroneal artery with no flow to the foot (Figure 1).

Covid-19 was diagnosed on the basis of reverse transcription polymerase chain reaction (RT-PCR) testing. Echocardiogram showed an ejection fraction of 60%. The patient was placed on air-borne precautions and was started on ceftriaxone, azithromycin, hydroxychloroquine, and therapeutic anticoagulation with heparin. He underwent thrombectomy of left common femoral artery, profunda femoris, superficial femoral artery, popliteal artery, anterior tibial artery, posterior tibial artery, and peroneal artery.

## Discussion

The clinical spectrum of COVID19 continues to evolve. The most common symptoms being reported are fever, myalgia, cough, and dyspnea, and less frequently headache, diarrhea, nausea, and vomiting [4]. Severity of infection could be varied from asymptomatic infection to critical disease. Smell and taste disorders (e.g., anosmia and dysgeusia) have also been reported as common symptoms in patients with COVID-19 [5]. Although in COVID-19 respiratory symptoms predominate, thrombosis can occur with COVID-19 [6]. Among patients with advanced age and medical comorbidities (cardiovascular disease, diabetes mellitus, hypertension, chronic lung disease, cancer, chronic kidney disease, obesity, and smoking), COVID-19 is frequently severe [7].

Patients with severe COVID-19 infection can develop a disseminated coagulopathy characterized by increases in procoagulant factors like fibrinogen and D-dimer leading to widespread microvascular thrombosis. This state has been termed thromboinflammation or COVID-19-associated coagulopathy [8]. Bellosta et al. evaluated 20 patients with ALI who were positive for COVID-19 and concluded that revascularization was lower than expected, which was hypothesized was due to a virus-related hypercoagulable state; however, the use of prolonged systemic heparin may improve surgical treatment efficacy as well as improve limb salvage and overall mortality [9]. Zhang et al. reported three COVID- 19 patients with cerebral infarcts. One of the patients had thrombosis in the bilateral lower limbs as well as in digits 2 and 3 of the left hand. In all the three cases, serologic tests were positive for anticardiolipin IgA antibodies as well as anti-β2-glycoprotein IgA and IgG antibodies. Lupus anticoagulant was not detected in any of the patients. These antibodies can arise transiently in patients with critical illness and various infections [10]. In our patient, antiphospholipid antibody testing was not done.

All hospitalized patients with COVID-19 should receive pharmacologic thromboprophylaxis with low molecular weight heparin (LMWH) or fondaparinux unless bleeding risk and full therapeutic-intensity anticoagulation in the appropriate clinical scenario [11]. Anticoagulant therapy mainly with LMWH appears to be associated with better prognosis in severe COVID-19 patients meeting sepsis-induced coagulopathy criteria or with markedly elevated D-dimer [12].

## Conclusions

We report an unusual presentation of a 77-year-old male patient who presented with dyspnea and acute pain in left leg and was found to have ALI and underwent thrombectomy. Our case and review of literature reveals that health care providers should be aware of this unusual life-threatening manifestation of COVID 19 so that appropriate measures can be taken for the vascular emergency. 
